# The interrater and intrarater reliability of the flexibility and strength tests included in the Sport Science Lab^®^ screening protocol amongst professional rugby players

**DOI:** 10.4102/sajp.v77i1.1504

**Published:** 2021-04-22

**Authors:** Candice MacMillan, Benita Olivier, Natalie Benjamin-Damons

**Affiliations:** 1 Department of Physiotherapy, Faculty of Health Sciences, University of the Witwatersrand, Johannesburg, South Africa

**Keywords:** rugby, injury risk factors, screening, reliability, manual strength testing

## Abstract

**Background:**

Considering the injury incidence rate (IR) associated with elite-level rugby, measures to reduce players’ injury risk are important. Establishing scientifically sound, pre-season musculoskeletal screening protocols forms part of injury prevention strategies.

**Objective:**

To determine the interrater and intrarater reliability of the flexibility and strength tests included in the Sport Science Lab^®^ (SSL) screening protocol.

**Methods:**

We determine the interrater and intrarater reliability of 11 flexibility and nine strength tests. Twenty-four injury-free, elite, adult (> 18 years), male rugby players were screened by two raters on two occasions. To establish intrarater and interrater reliability, Gwet’s AC_1_, AC_2_ and intraclass correlation coefficients (ICC) were used for the analysis of binary, ordinal and continuous variables, respectively. Statistical significance was set at 95%.

**Results:**

Flexibility tests which require lineal measurement had at least substantial interrater (ICC = 0.70–0.96) and intrarater reliability (ICC = 0.89–0.97). Most of the flexibility tests with binary outcomes attained almost perfect interrater and intrarater reliability (Gwet’s AC_1_ = 0.8–0.97). All strength tests attained at least substantial interrater (Gwet’s AC_2_ = 0.73–0.96) and intrarater (Gwet’s AC_2_ = 0.67–0.97) reliability.

**Conclusion:**

The level of interrater and intrarater reliability of most of the flexibility and strength tests investigated supports their use to quantify various aspects of neuromusculoskeletal qualities and possible intrinsic risk factors amongst elite rugby players.

**Clinical implications:**

Establishing the reliability of tests, is one step to support the inclusion thereof in official screening protocols. Results of our study, verify the reliability of the simple, clinically friendly strength and flexibility tests included and therefore support their use as preparticipation screening tools for rugby players. Further investigation as to the association thereof to athletes’ injury risk and performance is warranted.

## Introduction

Despite the high risk of injury related to the collisional nature of rugby union (henceforth rugby) (Schwellnus et al. 2014), the sport remains one of the most popular professional team sports worldwide (Brooks 2005). Rugby injury incidence rates (IR) have been considered high compared to sports such as soccer and basketball (Yeomans et al. 2018) but similar to other high-impact collisional sports such as Australian Rules football (Orchard & Seward 2002) and American National Football League (NFL) (Kerr et al. 2016).

Epidemiological studies conducted in South Africa (Schwellnus et al. 2014) and England (Brooks 2005) as well as a meta-analysis conducted by Williams et al. (2013) report similar findings regarding the incidence and nature of injury amongst professional male rugby players. All three studies concluded that the majority of injuries occur during matches (Injury IR 81.0–91.0 injuries/1000 player hours) and that injuries are related to a tackling incident. These studies also concurred that the injury rates for forwards and back-line players are similar and that the lower (48.1% – 58.9%) and upper limbs (15.6% – 25.6%) are the most common site of injuries.

The development of screening protocols has been advocated by various (Brooks 2005; Schwellnus et al. 2014; Van Mechelen, Hlobil & Kemper 1992) injury prevention paradigms. International rugby unions (Gray & Naylor 2012; Quarrie 2001) have developed pre-season musculoskeletal (MSK) screening protocols in an attempt to identify players at risk of sustaining in-season injuries. The protocol developed by the South African Rugby Union (SARU), which the developers claim to be similar to that of New Zealand and Australia, includes a series of physical screening tests related to, amongst others, strength, flexibility and joint range of motion (ROM) (Gray & Naylor 2012). Limited studies regarding the association of the tests included in these protocols and injury incidence amongst elite-level rugby players have however been published. Also, the developers’ rationale for inclusion of the tests was largely based on the tests’ reliability and normative values amongst athletes other than elite-level rugby players. Quarrie (2001) investigated various MSK performance measures amongst rugby players, of which only one was found to have a univariate relationship to injury. The developers of the Sport Science Lab^®^ (SSL) screening protocol therefore identified a need for evidence regarding existing MSK screening tools’ reliability and association with in-season injury. Hence, the aim of our study was to develop a screening protocol, investigate (amongst other qualities) the reliability thereof and publish the results based on the findings, and if necessary, amend the tool to improve the psychometric properties thereof.

When designing a screening protocol, the challenge lies in finding a delicate balance between scientific accuracy (reliability and validity) and practicality (ease and duration of execution; a small amount of inexpensive equipment and space, as well as the examination skill required) (Castro-Piñero et al. 2009). Reliability refers to the reproducibility of measurements within a given participant over time (intrarater reliability) and by various raters (interrater reliability) (Hayen, Dennis & Finch 2007). The ability of researchers to make inferences regarding certain outcome variables such as intrinsic risk factors is largely depended on repeated measurement accuracy, and the reliability of screening protocols is therefore pivotal (Dennis et al. 2008).

Xue (2016) suggested that better observer training, improved scale design and introducing items better at capturing heterogeneity improve the reliability of a screening tool. The developers considered both the proposed strategies to improve reliability (Xue 2016) and practicality thereof when designing the SSL screening protocol. The complete SSL screening protocol consists of 11 flexibility, seven strength, six plyometric and one rugby-specific fitness tests. As the plyometric and cardiorespiratory fitness tests are objective in nature (i.e. the raters do not have to measure, eyeball or base a rating on subjective measurement as is the case for the strength and flexibility tests), we did not include the plyometric and fitness tests in the reliability part of our study. The strength and flexibility tests included, equipment required and standard instructions are described in Online Appendix 1, Table 1-A1, whilst a detailed description of the purpose and rationale for the inclusion, modification of and proposed minimal standards for the flexibility and strength tests included in the protocol is summarised in Online Appendix 1, Table 2-A1.

### Rationale for inclusion of flexibility and strength tests, and manner of execution

Limitations in muscle flexibility and related joint mobility have been identified as injury risk factors amongst rugby players (O’Connor 2004; Yeomans et al. 2018). Considering the suggestions summarised by Xue (2016) regarding improvement of test reliability, flexibility tests were simplified to only include tape measured (lineal) outcomes or joint ROM, considered relative to stationary objects with either 0° horizontal or vertical planes such as a plinth.

### Rationale for inclusion of strength tests and manner of execution

The game of rugby requires players to tolerate and generate forces to propel their own and additional external weight loads. It is thus fair to regard muscular strength and power as important performance predictors (Posthumus & Durandt 2009) as well as intrinsic risk factors associated with injury prevention (Gamble 2004). Whilst strength doesn’t have a set definition or unit of measure, it is an attribute of force and power (Bohannon 2019). Manual muscle tests (MMT) have been used as a way to gauge muscle output (Bohannon 2019). The developers of the SSL screening protocol regarded MMT as the most practical option as they are inexpensive, quick and easily performed. The manner of execution and proposed rating scale is however new and has not been investigated. Some might argue that hand-held dynamometers (HHD) might be equally practical and provide more objective output measures. However, the cost of HHD may be prohibitive to some and the main limitation of MMT, that is, subjectivity of tester strength and related external resistance applied, is not overcome (Bohannon 2019). Further limitations of HHD and existing MMT strength rating scales are summarised in Online Appendix 1, Table 2-A1.

Our study is the first of two (the second investigates the association between the tests included in the protocol and in-season injury) conducted to establish a clinically useful, evidence-based, pre-season screening protocol that could be used by both medical and strength and conditioning professionals. In a team setting this would allow for a holistic picture of athletes’ pre-season intrinsic injury risks as well as to establish baseline fitness parameters. The aim of our study was thus to investigate the interrater and intrarater reliability of the SSL screening protocol.

## Methodology

This was a reliability study with a test–retest design. Guidelines for reporting reliability and agreement studies were followed (Kottner et al. 2011).

Information regarding our study was sent to 14 official national rugby unions requesting that they send a list of potential participants who volunteered. Participants included elite (i.e. part of an official SARU team) male rugby players between the ages of 19 and 36 years who were injury free at the start of the competitive rugby season. Players who were not on the active team roster at the start of the competitive rugby season were not eligible for inclusion. For convenience, the sample was selected based on the teams’/participants’ geographical proximity to the facility of an established sport rehabilitation and performance centre.

The sample size was calculated based on published guidelines regarding sample size requirements for two-rater reliability studies with nominal (Bujang & Baharum 2017; Sim & Wright 2005) or ordinal (Bujang & Baharum 2017) variables, which assume at least 50% positive ratings and a power of 80%. The authors of these studies suggest a sample size of between 25 (Sim & Wright 2005) and 29 (Bujang & Baharum 2017) participants. To account for dropout, 27 volunteers were included. Other similar reliability studies included 15 (O’Connor 2014) and 40 (Armstrong 2016) participants, respectively.

### Procedure

Our study commenced 3 weeks prior to the start of the competitive rugby season to allow for a standardised volume of training to have been completed. Intrarater and interrater reliability was assessed concurrently. The screening tests were conducted by a qualified physiotherapist (Rater 1; first author) and an athletic trainer (Rater 2). Both raters had more than 5 years of clinical experience and were experienced in the use of SSL screening protocol in daily practice. Two research assistants recorded the participants’ ratings/measurements. Raters were not allowed to communicate with each other during the rating of any of the screening tests and were blinded to the participants’ injury history and each other’s findings.

After performing a 10-min warm-up of their choice, participants were requested to perform all strength and flexibility tests as described in Appendices 1A and 1B. For time efficiency and minimal inconvenience to participants, all tests required to be done on the floor were done first (in no particular order), followed by the tests in standing and then tests performed on the plinth. Each test was performed three times and the best attempt was recorded.

Considering the logistics, practicality and training schedules of the participating teams, a week was dedicated to collect data. To minimise any physiological effects and allow symptoms that may have been provoked by the tests to subside, screening of participants occurred on two consecutive days, in the same environment, before training sessions. Ten participants were screened on two consecutive days and one day thereafter, and the remaining participants were screened on the next two consecutive days. During the screening sessions, each participant was screened once by Rater 1 and an hour later by Rater 2. To minimise potential recollection bias, the ordering of participants scheduled for a screening on a particular day, was randomised for each rater in both rating sessions. This randomisation, coupled with raters being blinded to ratings made during session 1, aimed to further reduce possible recollection bias.

### Data analysis

Statistical analyses were performed using Stata/IC 15.1 (StataCorp, TX, USA). Continuous variables were summarised by mean and standards deviation, whilst binary and ordinal variables were summarised by count and frequency.

Interrater reliability for both raters was determined by comparing per-session ratings (for both sessions) of Rater 1 with that of Rater 2. Intrarater reliability was analysed by comparing each rater’s day 1 ratings with that of day 2. To determine both interrater and intrarater reliability, Gwet’s AC_1_ (Gwet 2016) was used for tests with binary (yes or no) outcomes, Gwet’s AC_2_ (Gwet 2016) for ordinal variables and ICC_3,2_ (two-way mixed effects, consistency, multiple raters/measurements) (Mandrekar 2011) for tests with continuous outcome measures. The respective reliability coefficients with their 95% confidence intervals (CIs) were reported. Standard error of mean (SEM) values were also calculated. Intraclass correlation coefficient (ICC) values were interpreted according to the Landis and Koch scale (Landis & Koch 1977). Gwet’s agreement coefficients have been shown to be more stable and paradox-resistant (high percentage agreement but low *k*-value) than Cohen’s kappa (*k*) and other coefficients (Gwet 2014, 2016; Wongpakaran et al. 2013). Interpretation of results was done according to the benchmarking procedure as suggested by Gwet (2014), that is, the absolute agreement coefficients benchmarked as cumulative probability (in our case 95%), for any reliability coefficient to fall into one of the following categories: < 0.00, = Poor; 0.01–0.20 = Slight; 0.21–0.40 = Fair; 0.41–0.60 = Moderate; 0.61–0.80 = Substantial; 0.81–1.00 = Almost perfect. This method allows for direct and more precise comparisons of the different agreement coefficients and their representation on the Landis and Koch scale.

### Ethical considerations

Ethical approval was obtained from the University of the Witwatersrand Human Research Ethics Committee (Medical) (M180452). Written permission was obtained from the rugby union and the coaches of the respective teams and informed consent was obtained from players who volunteered to participate in our study.

## Results

Three (11.11%) of the participants (*n* = 27) did not attend the second screening session because of logistical problems or conflict with other obligations. Data for 24 participants were therefore analysed. The average age of the players was 19.96 (± 1.78) years, weight was 95.33 (± 13.50) kg and height was 186.50 (± 8.98) cm.

### Descriptive statistics

The descriptive statistics for flexibility tests with continuous outcomes are summarised in Table 1; flexibility tests with binary outcomes are summarised in Table 2 and all strength tests are summarised in Table 3. Considering the mean and minimum standards of the respective flexibility tests, both raters agreed on both days that most of the players did not achieve the minimum standards for the majority of tests. In contrast, the raters agreed that on both days the majority of players achieved the set minimum standards (score of 4 or 5) for most of the strength tests.

**TABLE 1:** Descriptive statistics for all strength and flexibility tests with continuous outcomes (*n* = 24).

Test	R1 D1			R2 D1			R1 D2			R2 D2		
	Mean	± SD	Range	Mean	± SD	Range	Mean	± SD	Range	Mean	± SD	Range
Ankle DF – R (cm)	10.08	± 2.78	2.00–15.00	10.83	± 2.66	4.00–15.00	10.00	± 2.38	4.00–15.00	10.83	± 2.88	5.00–16.00
Ankle DF – L (cm)	9.75	± 2.91	4.00–16.00	10.96	± 3.03	6.00–16.00	10.04	± 2.60	6.00–16.00	10.54	± 2.96	5.00–17.00
Toe-touch test (cm)	1.88	± 10.90	−14.00–37.00	−0.75	± 6.44	−12.00–38.00	2.04	± 10.74	−12.00–38.00	0.08	± 7.25	−13.00–16.00
V-sit (cm)	42.63	± 11.86	16.00–62.00	39.08	± 11.59	14.00–64.00	42.17	± 11.92	14.00–64.00	39.38	± 11.39	15.00–58.00
Combined shoulder flexion (cm)	32.96	± 10.95	13.00–56.00	36.63	± 12.09	15.00–55.00	32.88	± 10.96	15.00–55.00	35.00	± 10.41	17.00–53.00
Combined shoulder extension (cm)	37.17	± 10.52	14.00–50.00	41.33	± 9.68	20.00–55.00	38.25	± 10.30	20.00–55.00	41.42	± 8.56	20.00–60.00
Combined shoulder mobility – R (cm)	4.63	± 4.73	0.00–18.00	4.83	± 5.03	0.00–19.00	5.08	± 5.32	0.00–19.00	5.17	± 5.32	0.00–19.00
Combined shoulder mobility – L (cm)	3.92	± 0.83	0.00–19.00	3.92	± 0.93	0.00–18.00	3.88	± 0.85	0.00–18.00	3.88	± 0.95	0.00–21.00
Double leg lowers (repetitions)	19.04	± 5.81	6.00–23.00	18.21	± 7.05	4.00–23.00	18.38	± 6.11	4.00–23.00	18.63	± 6.11	5.00–23.00
Oblique twist (repetitions)	18.29	± 3.98	5.00–20.00	19.13	± 2.88	8.00–20.00	19.25	± 2.51	8.00–20.00	19.13	± 2.88	8.00–60.00

R, right; L, left; R1, rater1; R2, rater 2; D1, day 1; D2, day 2; DF, dorsiflexion; SD, standard deviation.

**TABLE 2:** Descriptive statistics for flexibility tests with binary outcomes (*n* = 24).

Test	Outcome	R1 D1		R2 D1		R1 D2		R2 D2	
		Count	%	Count	%	Count	%	Count	%
Ankle PF R	Yes	12.00	50.00	12.00	50.00	12.00	50.00	12.00	50.00
	No	12.00	50.00	12.00	50.00	12.00	50.00	12.00	50.00
Ankle PF L	Yes	11.00	45.83	12.00	50.00	11.00	45.83	11.00	45.83
	No	13.00	54.17	12.00	50.00	13.00	54.17	13.00	54.17
Hip IR R	Yes	24.00	100.00	23.00	95.83	24.00	100.00	23.00	95.83
	No	0.00	0.00	1.00	4.17	0.00	0.00	1.00	4.17
Hip IR L	Yes	23.00	95.83	22.00	91.67	23.00	95.83	22.00	91.67
	No	1.00	4.17	2.00	8.33	1.00	4.17	2.00	8.33
Hip ER R	Yes	13.00	54.17	18.00	75.00	10.00	41.67	16.00	66.67
	No	11	45.83	6.00	25.00	14.00	58.33	8.00	33.33
Hip ER L	Yes	9.00	37.50	17.00	70.83	9.00	37.50	16.00	66.67
	No	15.00	62.50	7.00	29.17	15.00	62.50	8.00	33.33
Thomas R – Psoas	Yes	9.00	37.50	16.00	66.67	10.00	41.67	16.00	66.67
	No	15.00	62.50	8.00	33.33	14.00	58.33	8.00	33.33
Thomas L – Psoas	Yes	8.00	33.33	13.00	54.17	11.00	45.83	12.00	50.00
	No	16.00	66.67	11.00	45.83	13.00	54.17	12.00	50.00
Thomas R – Rec. fem	Yes	8.00	33.33	10.00	42.00	11.00	45.83	12.00	50.00
	No	16.00	66.67	14.00	58.33	13.00	54.17	12.00	50.00
Thomas L – Rec. fem	Yes	6.00	25.00	11.00	45.83	8.00	33.33	10.00	41.67
	No	18.00	75.00	13.00	54.17	16.00	66.67	14.00	58.33
Thomas R – ITB	Yes	16.00	66.67	12.00	50.00	14.00	58.33	11.00	45.83
	No	8.00	33.33	12.00	50.00	10.00	41.67	13.00	54.17
Thomas L – ITB	Yes	12.00	50.00	14.00	58.33	13.00	54.17	17.00	70.83
	No	12.00	50.00	10.00	41.67	11.00	45.83	7.00	29.17
Shoulder rotation – R	Yes	23.00	95.83	23.00	95.83	23.00	95.83	23.00	95.83
	No	1.00	4.17	1.00	4.17	1.00	4.17	1.00	4.17
IR deficit – R	Yes	0.00	0.00	1.00	4.17	1.00	4.17	1.00	4.17
	No	24.00	100.00	23.00	95.83	23.00	95.83	23.00	95.83
ER deficit – R	Yes	0.00	0.00	0.00	0.00	0.00	0.00	0	0.00
	No	24.00	100.00	24.00	100.00	24.00	100.00	24.00	100.00
Shoulder rotation – L	Yes	23.00	95.83	24.00	100.00	23.00	95.83	24.00	100.00
	No	1.00	4.17	0.00	0.00	1.00	4.17	0	0.00
IR deficit – L	Yes	0.00	0.00	0.00	0.00	1.00	4.17	0	0.00
	No	24.00	100.00	24.00	100.00	23.00	95.83	24.00	100.00
ER deficit – L	Yes	1.00	4.17	0.00	0.00	0.00	0.00	0.00	0.00
	No	23.00	95.83	24.00	100.00	24.00	100.00	24.00	100.00

R, right; L, left; R1, rater1; R2, rater 2; D1, day 1; D2, day 2; DF, dorsiflexion; IR, internal rotation; ER, external rotation; ITB, iliotibial band; PF, plantar flexion; Rec. fem, rectus femoris muscle.

**TABLE 3:** Descriptive statistics for strength tests rated on a scale of 1–5† (*n* = 24).

Test	Rating†			5	4	3	2
	Rater	Day	Mode	%	%	%	%
Glut/ham R	R1	D1	3.00	29.17	33.33	37.50	0.00
	R2	D1	4.00	29.17	41.67	20.83	8.33
	R1	D2	4.00	25.00	41.67	29.17	4.17
	R2	D2	4.00	29.17	37.50	25.00	8.33
Glut/ham L	R1	D1	4.00	20.83	37.50	33.33	8.33
	R2	D1	4.00	29.17	37.50	29.17	4.17
	R1	D2	4.00	25.00	37.50	33.33	4.17
	R2	D2	5.00	37.50	29.17	29.17	4.17
Hip IR R	R1	D1	3.00	29.17	8.33	50.00	12.50
	R2	D1	3.00	33.33	16.67	37.50	12.50
	R1	D2	3.00	29.17	16.67	37.50	16.67
	R2	D2	3.00	33.33	16.67	37.50	12.50
Hip IR L	R1	D1	5.00	33.33	16.67	33.33	16.67
	R2	D1	5.00	33.33	20.83	33.33	12.50
	R1	D2	3.00	29.17	12.50	41.67	16.67
	R2	D2	5.00	29.17	25.00	25.00	20.83
HIP ER R	R1	D1	3.00	25.00	12.50	50.00	12.50
	R2	D1	3.00	29.17	12.50	50.00	8.33
	R1	D2	3.00	25.00	16.67	45.83	12.50
	R2	D2	3.00	29.17	20.83	41.67	8.33
Hip ER L	R1	D1	3.00	20.83	16.67	45.83	16.67
	R2	D1	3.00	29.17	16.67	41.67	12.50
	R1	D2	3.00	25.00	16.67	45.83	12.50
	R2	D2	3.00	29.17	20.83	41.67	8.33
Abduction R	R1	D1	5.00	45.83	41.67	12.50	0.00
	R2	D1	4.00	41.67	41.67	12.50	4.17
	R1	D2	4.00	45.83	50.00	4.17	0.00
	R2	D2	4.00	37.50	54.17	0.00	8.33
Abduction L	R1	D1	5.00	50.00	37.50	12.50	0.00
	R2	D1	4.00	37.50	45.83	12.50	4.17
	R1	D2	5.00	50.00	45.83	4.17	0.00
	R2	D2	4.00	37.50	54.17	8.33	0.00
Adduction R	R1	D1	5.00	79.17	12.50	8.33	0.00
	R2	D1	5.00	54.17	29.17	16.67	0.00
	R1	D2	5.00	83.33	4.17	12.50	0.00
	R2	D2	5.00	58.33	25.00	12.50	4.17
Adduction L	R1	D1	5.00	83.33	12.50	4.17	0.00
	R2	D1	5.00	58.33	25.00	16.67	0.00
	R1	D2	5.00	83.33	4.17	12.50	0.00
	R2	D2	5.00	62.50	16.67	20.83	0.00
Shoulder IR R	R1	D1	5.00	62.50	16.67	20.83	0.00
	R2	D1	5.00	58.33	25.00	16.67	0.00
	R1	D2	5.00	54.17	37.50	8.33	0.00
	R2	D2	5.00	54.17	37.50	8.33	0.00
Shoulder ER R	R1	D1	5.00	62.50	20.83	16.67	0.00
	R2	D1	5.00	50.00	33.33	16.67	0.00
	R1	D2	4.00	41.67	45.83	12.50	0.00
	R2	D2	4.00	37.50	45.83	12.50	4.17
Shoulder IR L	R1	D1	5.00	62.50	29.17	4.17	4.17
	R2	D1	5.00	54.17	41.67	4.17	0.00
	R1	D2	5.00	66.67	29.17	4.17	0.00
	R2	D2	4.00	50.00	50.00	0.00	0.00
Shoulder ER L	R1	D1	5.00	66.67	16.67	16.67	0.00
	R2	D1	5.00	58.33	29.17	12.50	0.00
	R1	D2	4.00	41.67	41.67	8.33	8.33
	R2	D2	4.00	33.33	54.17	12.50	0.00

R, right; L, left; R1, rater1; R2, rater 2; D1, day 1; D2, day 2; DF, dorsiflexion; IR, internal rotation; ER, external rotation; Glut/ham, gluteus maximus/hamstring.

† , None of the raters gave a rating of 1 to any of the participants for any of the tests, the percent frequency for a rating of 1 is therefore not summarised in the table.

### Inter- and intrarater reliability

The inter- and intrarater agreement coefficients, CI and standard error (SE) for flexibility tests with continuous outcomes are summarised in Table 4; flexibility tests with binary outcomes in Table 5 and strength tests in Table 6.

**TABLE 4:** Inter- and intrarater reliability for flexibility tests with continuous outcomes.

Test	Interrater reliability					Intrarater reliability				
	Day	ICC_3,2_	95% CI	SEM	Interpretation†	Rater	ICC_3,2_	95% CI	SEM	Interpretation†
Ankle DF – R (cm)	D1	0.95	0.88–0.98	2.28	Almost perfect	R1	0.97	0.94–0.99	1.59	Almost perfect
	D2	0.96	0.91–0.98	1.97	Almost perfect	R2	0.96	0.91–0.98	2.04	Almost perfect
Ankle DF – L (cm)	D1	0.95	0.89–0.98	2.24	Almost perfect	R1	0.99	0.97–0.99	1.14	Almost perfect
	D2	0.95	0.90–0.98	2.10	Almost perfect	R2	0.95	0.90–0.98	2.27	Almost perfect
Toe-touch test (cm)	D1	0.70	0.40–0.86	8.24	Substantial	R1	0.98	0.95–0.99	3.00	Almost perfect
	D2	0.70	0.40–0.86	8.55	Substantial	R2	0.89	0.77–0.95	3.92	Almost perfect
Low back V-Sit (cm)	D1	0.98	0.95–0.99	6.12	Almost perfect	R1	0.99	0.97–0.99	5.11	Almost perfect
	D2	0.97	0.94–0.99	6.74	Almost perfect	R2	0.98	0.96–0.99	5.40	Almost perfect
Combined shoulder flexion (cm)	D1	0.90	0.77–0.95	11.28	Almost perfect	R1	0.93	0.84–0.97	8.56	Almost perfect
	D2	0.94	0.85–0.98	8.43	Almost perfect	R2	0.93	0.85–0.97	9.52	Almost perfect
Combined shoulder extension (cm)	D1	0.92	0.83–0.97	10.45	Almost perfect	R1	0.95	0.90–0.98	8.48	Almost perfect
	D2	0.95	0.90–0.98	8.48	Almost perfect	R2	0.97	0.93–0.99	6.87	Almost perfect
Combined shoulder mobility – R (cm)	D1	0.94	0.87–0.97	2.39	Almost perfect	R1	0.95	0.88–0.98	1.41	Almost perfect
	D2	0.98	0.95–0.99	1.67	Almost perfect	R2	0.98	0.96–0.99	1.41	Almost perfect
Combined shoulder mobility – L (cm)	D1	0.93	0.84–0.97	3.41	Almost perfect	R1	0.96	0.91–0.98	2.38	Almost perfect
	D2	0.91	0.80–0.96	3.85	Almost perfect	R2	0.96	0.91–0.98	2.38	Almost perfect

D, day; R, rater; ICC, intra-class correlation coefficient; SEM, standard error measurement; CI, confidence interval; R, right; L, left; DF, dorsiflexion.

† , Landis and Koch scale; Gwet’s probabilistic benchmarking.

**TABLE 5:** Inter- and intrarater reliability for flexibility tests with binary outcomes.

Test	Interrater reliability				Interpretation†	Intrarater reliability				Interpretation†
	Day	Gwet AC_1_	95% CI	SE		Rater	Gwet AC_1_	95% CI	SE	
Ankle Plantar Flexion – R	D1	0.83	0.60–1.00	0.11	Almost perfect	R1	1.00	1.00–1.00	0.00	Almost perfect
	D2	0.83	0.60–1.00	0.11	Almost perfect	R2	1.00	1.00–1.00	0.00	Almost perfect
Ankle Plantar Flexion – L	D1	0.75	0.47–1.00	0.14	Substantial	R1	0.83	0.60–1.00	0.12	Almost perfect
	D2	0.83	0.60–1.00	0.12	Almost perfect	R2	0.92	0.75–1.00	0.08	Almost perfect
Hip IR R	D1	0.96	0.00–0.86	0.05	Almost perfect	R1	1.00	1.00–1.00	0.00	Almost perfect
	D2	0.96	0.00–0.86	0.05	Almost perfect	R2	1.00	1.00–1.00	0.00	Almost perfect
Hip IR L	D1	1.00	0.95–0.99	0.00	Almost perfect	R1	1.00	1.00–1.00	0.00	Almost perfect
	D2	0.65	0.94–0.99	0.33	Substantial	R2	1.00	1.00–1.00	0.00	Almost perfect
Hip ER R	D1	0.46	0.07–0.86	0.19	Moderate	R1	0.58	0.23–0.93	0.17	Moderate
	D2	0.50	0.13–0.88	0.18	Moderate	R2	0.72	0.42–1.00	0.14	Substantial
Hip ER L	D1	0.46	0.07–0.86	0.19	Moderate	R1	0.58	0.23–0.93	0.12	Moderate
	D2	0.50	0.13–0.88	8.48	Moderate	R2	0.72	0.67–0.99	0.19	Substantial
Thomas R – Psoas	D1	0.25	−0.17–0.67	0.20	Fair	R1	0.76	0.48–1.00	0.69	Substantial
	D2	0.34	−0.07–0.75	0.20	Fair	R2	0.70	0.27–0.99	0.15	Substantial
Thomas R – Rec Fem	D1	0.22	−0.23–0.66	0.22	Fair	R1	0.60	0.25–0.95	0.17	Substantial
	D2	0.25	−0.17–0.67	0.20	Fair	R2	0.33	−0.07–0.73	0.20	Fair
Thomas R – ITB	D1	0.03	−0.43–0.41	0.22	Poor	R1	0.53	0.16–0.90	0.18	Moderate
	D2	0.58	0.23–0.93	0.17	Substantial	R2	0.25	−0.17–0.67	0.20	Fair
Thomas L – Psoas	D1	0.26	−0.17–0.68	0.68	Substantial	R1	0.60	0.25–0.95	0.17	Substantial
	D2	0.58	0.25–0.93	0.17	Substantial	R2	0.75	0.47–1.00	0.14	Substantial
Thomas L – Rec. fem	D1	0.16	−0.31–0.62	0.22	Slight	R1	0.72	0.42–1.00	0.14	Substantial
	D2	0.22	−0.23–0.66	0.22	Fair	R2	0.59	0.24–0.94	0.17	Moderate
Thomas L – ITB	D1	0.34	−0.70–0.75	0.19	Fair	R1	0.75	0.47–1.00	0.14	Substantial
	D2	0.38	−0.04–0.79	0.18	Fair	R2	0.46	0.07–0.86	0.19	Moderate
Combined shoulder rotation – R	D1	0.91	0.77–1.00	0.07	Almost perfect	R1	0.91	0.77–1.00	0.07	Almost perfect
	D2	1.00	1.00–1.00	0.00	Almost perfect	R2	1.00	1.00–1.00	0.00	Almost perfect
Shoulder IR deficit – R	D1	0.96	0.86–1.00	0.05	Almost perfect	R1	0.96	0.86–1.00	0.05	Almost perfect
	D2	1.00	1.00–1.00	0.00	Almost perfect	R2	1.00	1.00–1.00	0.00	Almost perfect
Shoulder ER deficit – R	D1	0.96	0.86–1.00	0.05	Almost perfect	R1	0.96	0.86–1.00	0.05	Almost perfect
	D2	1.00	1.00–1.00	0.00	Almost perfect	R2	1.00	1.00–1.00	0.00	Almost perfect
Combined shoulder rotation – L	D1	0.96	0.86–1.00	0.05	Almost perfect	R1	0.96	0.86–1.00	0.05	Almost perfect
	D2	0.96	0.86–1.00	0.05	Almost perfect	R2	1.00	1.00–1.00	0.00	Almost perfect
Shoulder IR deficit – L	D1	1.00	1.00–1.00	0.00	Almost perfect	R1	0.96	0.86–1.00	0.05	Almost perfect
	D2	0.96	0.86–1.00	0.05	Almost perfect	R2	1.00	1.00–1.00	0.00	Almost perfect
Shoulder ER deficit – L	D1	1.00	1.00–1.00	0.00	Almost perfect	R1	0.96	0.86–1.00	0.00	Almost perfect
	D2	1.00	1.00–1.00	0.00	Almost perfect	R2	1.00	1.00–1.00	0.00	Almost perfect

D, day; R, rater; SEM, standard error; CI, confidence interval R, right; L, left; ITB, iliotibial band; IR, internal rotation; ER, external rotation.

† , Landis and Koch scale; Gwet’s probabilistic benchmarking.

**TABLE 6a:** Intrarater and interrater reliability for all strength tests.

Test	Interrater reliability					Intrarater reliability				
	Day	Gwet’s AC_2_	CI	SE	Interpretation†	Rater	Gwet’s AC_2_	CI	SE	Interpretation†
Glut/Ham R	D1	0.92	0.85–1.00	0.04	Almost perfect	R1	0.95	0.88–1.00	0.03	Almost perfect
	D2	0.92	0.85–1.00	0.04	Almost perfect	R2	0.92	0.88–1.00	0.04	Almost perfect
Glut/Ham L	D1	0.87	0.77–0.96	0.04	Almost perfect	R1	0.94	0.88–1.00	0.03	Almost perfect
	D2	0.89	0.80–0.97	0.04	Almost perfect	R2	0.92	0.85–1.00	0.04	Almost perfect
Hip IR R	D1	0.91	0.82–0.99	0.04	Almost perfect	R1	0.91	0.82–0.99	0.04	Almost perfect
	D2	0.78	0.60–0.96	0.09	Substantial	R2	1.00	1.00–1.00	0.00	Almost perfect
Hip IR L	D1	0.87	0.71–1.00	0.07	Almost perfect	R1	0.88	0.74–1.00	0.07	Almost perfect
	D2	0.85	0.70–1.00	0.07	Almost perfect	R2	0.83	0.68–0.98	0.07	Almost perfect
Hip ER R	D1	0.95	0.88–1.00	0.03	Almost perfect	R1	0.95	0.88–1.00	0.03	Almost perfect
	D2	0.87	0.73–1.00	0.06	Almost perfect	R2	0.96	0.91–1.00	0.03	Almost perfect
Hip ER L	D1	0.87	0.76–0.97	0.05	Almost perfect	R1	0.87	0.77–0.97	0.05	Almost perfect
	D2	0.83	0.69–0.97	0.06	Almost perfect	R2	0.92	0.85–1.00	0.04	Almost perfect
Abduction R	D1	0.84	0.62–1.00	0.10	Almost perfect	R1	0.94	0.85–1.00	0.04	Almost perfect
	D2	0.89	0.70–1.00	0.09	Almost perfect	R2	0.91	0.83–0.99	0.04	Almost perfect
Abduction L	D1	0.81	0.59–1.00	0.10	Almost perfect	R1	0.94	0.85–1.00	0.04	Almost perfect
	D2	0.85	0.65–1.00	0.10	Almost perfect	R2	0.92	0.85–1.00	0.04	Almost perfect
Adduction R	D1	0.96	0.90–1.00	0.03	Almost perfect	R1	0.96	0.90–1.00	0.03	Almost perfect
	D2	0.83	0.64–1.00	0.09	Almost perfect	R2	0.97	0.92–1.00	0.02	Almost perfect
Adduction L	D1	0.73	0.48–0.99	0.12	Substantial	R1	0.96	0.90–1.00	0.03	Almost perfect
	D2	0.79	0.56–1.00	0.11	Substantial	R2	0.88	0.76–1.00	0.06	Almost perfect
Shoulder IR R	D1	0.88	0.76–1.00	0.06	Almost perfect	R1	0.67	0.37–0.97	0.14	Substantial
	D2	0.86	0.79–0.98	0.06	Almost perfect	R2	0.74	0.50–0.97	0.11	Substantial
Shoulder ER R	D1	0.85	0.71–0.98	0.07	Almost perfect	R1	0.71	0.46–0.97	0.12	Substantial
	D2	0.87	0.75–1.00	0.06	Almost perfect	R2	0.77	0.60–0.94	0.08	Substantial
Shoulder IR L	D1	0.91	0.80–1.00	0.05	Almost perfect	R1	0.85	0.67–1.00	0.09	Almost perfect
	D2	0.81	0.68–0.95	0.07	Almost perfect	R2	0.84	0.71–0.96	0.06	Almost perfect
Shoulder ER L	D1	0.80	0.65–0.96	0.08	Almost perfect	R1	0.80	0.58–1.00	0.10	Almost perfect
	D2	0.81	0.62–0.99	0.09	Almost perfect	R2	0.68	0.51–0.85	0.08	Substantial

D, day; R, rater; SE, standard error; CI, confidence interval; R, right; L, left; ITB, iliotibial band; IR, internal rotation; ER, external rotation; Glut/Ham, gluteus maximus/hamstring.

† , Landis & Koch scale; Gwet’s probabilistic benchmarking.

**TABLE 6b:** Intrarater and interrater reliability for all strength tests.

Test	Interrater reliability					Intrarater reliability				
	Day	ICC	CI	SE	Interpretation†	Rater	ICC	CI	SE	Interpretation†
Abdominals	D1	0.89	0.77–0.95	6.19	Almost perfect	R1	0.95	0.88–0.98	3.77	Almost perfect
	D2	0.93	0.91–0.98	4.96	Almost perfect	R2	0.87	0.71–0.95	5.88	Almost perfect
Obliques	D1	0.90	0.79–0.95	5.31	Almost perfect	R1	0.96	0.92–0.98	3.33	Almost perfect
	D2	0.97	0.95–0.99	2.61	Almost perfect	R2	0.92	0.82–0.96	4.89	Fair

D, day; R, rater; SE, standard error; CI, confidence interval; R, right; ICC, intraclass correlation coefficient.

† , Landis & Koch scale; Gwet’s probabilistic benchmarking.

### Flexibility tests

With the exception of the Toe Touch (TT) test, all other flexibility tests with continuous outcomes had almost perfect intrarater (ICC = 0.91–0.98) and interrater (ICC = 0.0.89–0.99) agreement. The TT test had substantial interrater agreement for both sessions and almost perfect intrarater agreement.

Except for the Modified Thomas test (MTT) and hip ER tests, all binominal flexibility tests had at least substantial inter- and intrarater reliability (Gwet AC_1_ = 0.65–1.00; SE < 0.12). Interrater reliability for all aspects of the Thomas test (i.e. psoas, rectus femoris and ITB) on both sides were at most moderate, with Gwet’s AC_1_, respectively, ranging from 0.22 to 0.58, 0.16 to 0.22, and 0.03 to 0.38. Intrarrater reliability for the Thomas tests ranged from slight to substantial (Gwet’s AC_1_ = 0.25–0.76), with larger CI compared to other binary tests. Notably, the intrarater reliability for Rater 1 was consistently higher than that of Rater 2.

### Strength tests

All strength tests had at least substantial interrater (Gwet’s AC = 0.73–0.96) and intrarater (Gwet’s AC_2_ = 0.67–0.96) agreement with small SE (< 0.15). The abdominal and oblique strength tests had almost perfect intrarater (ICC = 0.90–0.96) and interrater agreement (ICC = 0.77–0.92) with small SE (SE = 2.61–6.19) compared to the test means as summarised in Table 1.

## Discussion

Because of the collisional nature of rugby, injuries seem an inevitable part of the game. However, clinicians should continuously seek strategies to minimise the incidence and severity of injuries. For medical and conditioning staff involved in elite-level sports, such strategies involve the development of practical and scientifically sound pre-season MSK screening protocols to identify possible intrinsic risk factors to injury.

Like Ashworth et al. (2018) who investigated the reliability of an original upper body strength test, our study only included elite adult male rugby players. The anthropometrics and demographics (age) of the players in our study were similar to that of Ashworth et al. (2018). Haitz et al. (2014) investigated the inter- and intrarater reliability of a battery of screening tests amongst collegiate athletes (i.e. all participating at the same level) of various sports and reported high levels of inter-rater (*k* = 0.83–1.00) and intrarater (*k* = 0.71–0.95) reliability. A degree of homogeneity in the level of participation and sporting activity might therefore have a significant impact on the outcomes of studies investigating the reliability of neuromusculoskeletal screening tests. One of the reasons is that elite athletes’ ability to recover after performing multiple physical fitness tests exceeds that of athletes participating at lower levels of competition. The variability in test results, because of the possible physiological effects of repeated physical fitness testing (more specifically strength and flexibility), by multiple raters on multiple days, may therefore be more limited and more reliable.

Considering the mean of the toe-touch test (TT test) (–0.75 cm – 2.04 cm), the standard deviation (SD) (4.73–5.32) was large. The TT test is the only test of which the outcome distribution is bimodal (i.e. outcomes can be both, greater or less than zero). The large SD can therefore be explained by the cumulative, mathematical effect of including both positive and negative values in the calculation of the mean and in turn SD. The calculation of SEM takes SD and ICC into account. Considering the high SD (in addition to the lower interrater reliability ICC: [0.70 {0.40–0.86}]) values, it is not surprising that the interrater SEM (8.24–8.55) for the TT test was also high. The SD for the combined right shoulder mobility test was also large, considering the mean (mean = 4.63 cm – 5.17 cm; SD = 4.73 cm – 5.32 cm). This could be attributed to the number of zero measurements included in the data set.

All lineal flexibility tests had at least substantial interrater reliability (0.70–0.98) and almost perfect intrarrater reliability (0.89–0.98) and, except for the TT test, had small corresponding CI as well. This can be attributed to the objective, simple precision with which outcomes can be measured using a tape measure. Although, interrater reliability of the TT did not achieve the acceptable benchmarks set by the authors (i.e. almost perfect), the intrarater reliability did achieve the acceptable standards. Interrater reliability for this test can be improved by a more thorough description of the test, specifically ensuring that raters identify all possible compensatory mechanisms related to achieving better test scores, for example, by slightly bending the knees.

Although the TT, combined shoulder flexion and extension, and v-sit tests had at least substantial inter- and intrarater reliability, their respective SEMs were larger than other lineal flexibility tests. At first glance, it may seem that these values are indicative of a lesser degree of agreement. However, this can be attributed to the larger range of possible scores (i.e. greater distribution range) associated with the respective tests. For example, the maximum range for ankle DF might be limited from 0 cm to 20 cm where combined shoulder flexion and extension has an outcome range of 0 cm to > 60 cm. For larger range outcomes the variability (i.e. SD) may be more extensive, resulting in larger SEM values.

Most flexibility tests with binary outcomes attained almost perfect intrarater and interrater reliability (Gwet’s AC_1_ > 0.8). The MTT and hip ER tests yielded lower intrarater and interrater reliability values (Gwet’s AC < 0.73). The difference in the reliability achieved for these tests can be attributed to the complexity of the tests. Whilst tests that require the observation of single joint movement or for which the rating criteria is obvious (e.g. dorsal aspect of the foot and ankle has to be flat against the floor), the MTT and hip ER tests challenge the flexibility and range of multiple joints and structures simultaneously, thereby making the rating criteria more complicated. Numerous studies investigating the reliability of observational neuromusculoskeletal tests that require assessment of more than one component have been found to have poor intrarater reliability (Monnier et al. 2012; Moreland et al. 1997; Whatman, Hume & Hing 2015). To improve reliability, one can consider simplifying the tests by executing, for example, the MTT three times and only assessing one aspect per repetition. Another consideration is to measure the outcomes of the test more objectively, using a goniometer. However, Peeler and Anderson (2007) reported poor interrater and intrarater reliability regardless of whether an observational dichotomous (fail/pass) scale or goniometer was used for measurement of the various aspects of the Thomas test. The hip ER test might be improved by the objective measurement of the linear distance of the forehead to plinth surface using a tape measure. If the participant is unable to place the lateral aspect of the test leg knee flat on the plinth, the distance from the lateral epicondyle to plinth surface can also be measured as a baseline for tracking progress.

Several MMT’s and rating scales have been documented (Avers & Brown 2019; Cuthbert & Goodheart 2007). However, some have fundamental shortcomings when applied to an athletic population. The main limitations related to their relevance in a rugby population, as explained in the introduction and Online Appendix 1, Table 2-A1, are related to non-functional player position during testing and the type of muscle actions (concentric only) tested. The manual strength testing regime proposed by the developers of the SSL screening protocol attempts to address some of the shortcomings of existing manual strength testing regimes.

Considering the physicality of MMT, the subjectivity of tester resistance and tester strength have been identified as factors limiting the reliability thereof, particularly amongst higher scores (Bohannon 2019). In our study, the anthropometrics and demographics of the raters differed vastly (Rater 1 – Female, 34 years, height = 168 cm, weight = 60.00 kg; Rater 2 – Male, 28 years, height = 188 cm, weight, 100 kg), yet the interrater reliability of all manual strength tests, with the exception of the left hip adduction which was substantial, were almost perfect (Gwet’s AC_2_ = 0.81–0.96; SE [0.03–0.12]). The level of reliability and agreement therefore did not seem to be affected by the raters’ physical characteristics or resistance-related subjectivity. In fact, perhaps contrary to what one would expect, considering the modes in Table 3, Rater 1 rated most players’ strength lower than Rater 2 on two occasions (day 1 – right glut/ham; day 2 glut/ham; day 2-left hip IR) and the same as Rater 1 for 21 (out of 28) occasions.

The modes further indicate that, with the exception of the right glut/hamstring and left hip IR tests, both raters on both days agreed that the majority of the participants met or did not meet the proposed minimum standards. It therefore appears that both raters had similar clinical decision-making skills, reiterating the importance of well-described testing procedures and adequate training in the use of the tools. Specifically, the testers’ understanding of the position and hand placement that allows for optimal biomechanical advantage when the external force is applied is crucial.

Reliability studies investigating MMT amongst elite, healthy athletes are rare. Manual muscles tests (MMTs), such as the ‘break-test’ (Avers & Brown 2019), have good reliability for assessing individuals with neuromusculoskeletal dysfunction (Cuthbert & Goodheart 2007). In our study, the authors proposed the use of a novel MMT strength test battery and rating scale for screening, as opposed to a diagnostic tool, for asymptomatic, seemingly healthy individuals. Manual muscles tests evaluate the ability of the nervous system to adapt to either meet or counter the changing pressure exerted by the examiner (Cuthbert & Goodheart 2007). The developers of the SSL strength testing regime therefore assume that an optimal functioning, well-trained nervous system will immediately alter motor unit recruitment in an attempt to meet the demands of the test (external pressure/force applied), whilst sub-optimal or a dysfunctional nervous system, or structurally damaged muscle fibres, that they innervate, will fail to do so.

Cuthbert and Goodheart (2007) reported that studies investigating the level of agreement for MMT amongst symptomatic or asymptomatic, non-sporting participants attained high levels of interrater (82.00% – 97.00%) and intrarater (96.00% – 98.00%) agreement. Similarly, we found substantial agreement between raters (Gwet’s AC_2_ = 0.73–0.96) and sessions (Gwet’s AC_2_ = 0.67–0.96) for MMT executed and rated according to the SSL guidelines. The strength tests, based on the number of repetitions completed, that is, the double leg lower and oblique twist tests, also yielded high interrater (ICC = 0.89–0.93 and 0.90–0.99, respectively) and intrarater (ICC = 0.90–0.96 and 0.92–0.96) reliability.

### Limitations and strengths of the study

The reliability measures were based on the fixed raters (not randomly selected) who participated in our study, and the results may be limited to this specific group of raters. Only elite adult male rugby players were investigated and the results are therefore not generalisable to other sports, or youth players and/or players playing at a different level. Although a power analysis was done, the sample size was small. Further research is required with larger cohorts. Ideally, if the team’s schedules allowed, a longer wash-out period would have been introduced to further reduce recollection bias. The strength of our study is that a homogeneous population, following the same training schedule, was evaluated. Therefore, the variability of individualised scores because of physiological changes arising from testing or training (and other possible confounding variables such as training load between sessions) was limited.

### Clinical and research implications

Reliability of screening protocols is essential as it is of fundamental importance to the quality of players’ healthcare and performance, so that the professionals can replicate and agree on their findings and conclusions. Furthermore, reliable tools should reflect the qualities of the group of participants being screened and not the raters involved in the screening. Raters involved in our study had experience in the use of the SSL screening protocol, emphasising the importance of raters being trained in the use of standardised protocols. Future studies should focus on establishing the reliability of this screening protocol amongst novice raters with less experience, across a range of different sporting professionals as well as amongst athletes participating at different levels and in other sports. As the reliability of most of the tests included in the SSL protocol has been established, the association of these tests with injury risk could be investigated to establish players’ injury risk profiles at the start of the season and in turn develop targeted injury prevention strategies.

Knotter and Steiner (2011) emphasised that the difference in ratings is not solely a statistical decision, but also a clinical one. In clinical practice, the interpretation should consider that the purpose and consequences of the test results are to establish the acceptable margin of error for clinical decision-making. Here, like in other studies (Knotter & Steiner 2011), unless there were statistically sound reasons for lower reliability coefficient values, we considered values of at least 0.80 (i.e. ‘perfect agreement’) as clinically acceptable. Lower values might however still be useful for research purposes and group comparisons (Kottner et al. 2011).

## Conclusion

Most of the flexibility and strength tests included in the SSL screening protocol demonstrated at least substantial intrarater and interrater reliability. Establishing the reliability of this protocol is one step closer to support its use as a clinical tool to quantify various aspects of neuromusculoskeletal qualities and identify possible intrinsic risk factors amongst adult, elite male rugby players. Additionally, the test results reported here can provide baseline scores or measurements for comparison with similar or different level athletes. Continued efforts should be made by the developers of the SSL screening protocol to improve the reliability, or include alternative tests to assess the hip flexor and external rotation ROM.
